# Walsh’s Physician’s Call-Book and Tablet

**Published:** 1876-11

**Authors:** 


WALSH'S PHYSICIANS' COMBINED CALL-BOOK AND TABLET.—The book is of convenient size for pocket memoranda, and contains various tables, such as "regulating the dose of medicine for children, apothecaries' abreviations, poisons and their antidotes, formulæ and doses of medicines for hypodermic injections, metrical weights and measures, etc.; also chemical tests of urine, directions for post-mortem examinations, treatment of asphyxia, etc.

The call-list, which serves the purpose of recording engagements as well as calls made, is interleaved so as to furnish for each week a blank page suited to remarks, notes of cases, charges, etc. Physicians once in the habit of using such books, find great inconvenience in the practice of medicine without one.

Price, $1.50. For sale by Lippincott & Co., and book-sellers generally.

